# Development of Nested PCR-Heteroduplex Mobility Assay for Determination of Genetic Diversity in the Block 2 Region of the *Plasmodium falciparum* Merozoite Surface Protein 1 Gene

**DOI:** 10.1155/2020/9520326

**Published:** 2020-04-08

**Authors:** Kanyanan Kritsiriwuthinan, Warunee Ngrenngarmlert, Sakone Sunantaraporn, Anna Jehmah

**Affiliations:** ^1^Faculty of Medical Technology, Rangsit University, Pathumthani, Thailand 12000; ^2^Faculty of Medical Technology, Mahidol University, Nakhon Pathom, Thailand 73170; ^3^Vector Biology and Vector Borne Disease Research Unit, Department of Parasitology, Faculty of Medicine, Chulalongkorn University, Bangkok, Thailand 10330; ^4^Maelan Hospital, Pattani, Thailand 94180

## Abstract

Genetic diversity of *Plasmodium* parasite has significantly related to malaria control and vaccine development. The *P. falciparum* merozoite surface protein 1 (*Pfmsp1*) gene is a commonly used molecular marker to differentiate genetic diversity. This study is aimed at developing a nested PCR-Heteroduplex Mobility Assay (nPCR-HMA) for determination of the block 2 of the *Pfmsp1* gene. The MAD20 family allele of *P. falciparum* was used as a control for optimization of the annealing and polyacrylamide gel electrophoresis conditions. In order to evaluate the developed nPCR-HMA, 8 clinical *P. falciparum* isolates were examined for allelic variants. The results revealed 9 allelic variants. Our study indicated that the successful nPCR-HMA with good precision and accuracy offers a more rapid, efficient, and cheap method for large-scale molecular epidemiological studies as compared to nucleotide sequencing.

## 1. Background

Malaria is a severe infectious disease, caused by the parasites which belongs to the *Plasmodium* genus and transmits to humans by the female anopheles mosquito vector. It is a significant public health problem worldwide, especially in tropical and subtropical regions. Regardless of widespread efforts to eradicate or control malaria, this disease continues as the leading cause of morbidity and mortality [1]. In 2017, around 219 million people are at risk of malaria and the estimated number of malaria deaths accounted for 435,000 [1].

Human malaria is caused by five species including *P. falciparum*, *P. vivax*, *P. malariae*, *P. ovale*, *and P. knowlesi*. Among these, *P. falciparum* is the most widespread and the most dangerous. *P. falciparum* responsible for 99.7% of estimated malaria cases in the WHO African region, as well as in the majority of cases in the WHO regions of South-East Asia (62.8%), the Eastern Mediterranean (69%), and the Western Pacific (71.9%) [1].

The spread and mortality rate of malaria infection associate to several features. The increasing of the immigration of foreign workers from endemic areas into countries can lead to the spread of malaria between regions [2–5]. In addition, the genetic variation has been reported as a cause of the mechanisms that escape from the immune response of the host, and the increasing resistance to the antimalarial drugs [6–9] also is a cause of malaria spreading.

The genotyping of malaria parasite populations is used to investigate the genetic diversity of malaria infections with consideration of several factors, including transmission intensity, drug-resistant parasite, precise treatment, host immunity, and epidemiologic study. Thus, simple and accurate genetic diversity detection has developed. Several molecular techniques have been used for genetic diversity detection, including nested PCR, Restriction Fragment Length Polymorphism (RFLP), Single-Strand Conformation Polymorphism (SSCP), Heteroduplex Tracking Assay (HTA), Heteroduplex Mobility Assay (HMA), and DNA sequencing. The HMA has been used for subtyping, identification, and classification of human immunodeficiency virus (HIV) and other viruses [10–14]. The HMA is a simple, rapid, sensitive, and cost-effective technique compared to DNA sequencing [10].

The principle of HMA is based on a reference DNA and a sample DNA that will be amplified separately, mixed, denatured, and slowly cooled to permit the formation of heteroduplex from incomplete base matching between reference DNA and sample DNA. Then, the heteroduplex is determined by using polyacrylamide gel electrophoresis. The differences in the migration of heteroduplex form homoduplex indicate the difference in nucleotide sequence between reference DNA and a sample DNA. These are due to mismatched regions which present single-stranded configuration and slower migration than completed match dsDNA. In this system, if the sample DNA is not a perfect match to the reference DNA, multiple bands will be observed. The fastest migrating band is the homoduplex reference DNA and/or the homoduplex DNA sample. A heteroduplex band with mismatches migrates more slowly than a homoduplex band. HMA is easily applied to large numbers of samples and is particularly suited to the analysis of PCR products, because both the sample DNA and the reference DNA must be of the same size. In PCR, this would correspond to the use of the same amplification primers.

In principle, HMA shows the characteristics of 3 bands: the first band is a fast DNA moving as a homoduplex band caused by a complete sequence matching self DNA or a complete sequence matching in the sample DNA. The second band was the slowest-moving band caused by an incomplete match between 2 DNA sequences. The last band is a single-stranded DNA that occurs in the annealing step that has a matching DNA with the reference DNA independent of the sample; reducing the temperature to minimize single partial DNA matching thus forms a single-stranded DNA band.

The *P. falciparum* merozoite surface protein 1 (*Pfmsp1*) gene is typically used as a genetic marker to determine the genetic diversity and multiplicity of infection (MOI) of *P. falciparum* [15–17]. *PfMSP1* located on chromosome 9 contains 17 blocks, of which block 2 shows prevalent allelic polymorphism worldwide [16, 17]. The block 2 allele is mainly represented by three families, namely, K1, MAD20, and RO33 in the field isolates. These families' allelic sequences show a highly diverse and specific geographical distribution [18]. In addition, the pattern and fragment size polymorphism of the block 2 allele serve as molecular indicators, host immunity, and malaria transmission dynamics [19].

However, data on the HMA to investigate the genetic diversity of the *Pfmsp1* gene have not been evaluated. In this study, we developed and evaluated the Nested PCR-Heteroduplex Mobility Assay (nPCR-HMA) to determine the genetic diversity of the *Pfmsp1* gene in the block 2 region. The technique could be used for the screening of *P. falciparum* genetic diversity, reinfection, recrudescence, and molecular epidemiology study in a malaria-endemic area.

## 2. Material and Methods

### 2.1. Sample Collection

The study was approved and reviewed by the Ethics Committee of Rangsit University, Thailand (RSUERB2019-061). The samples tested composed of 8 *P. falciparum*-infected blood detected by microscopic examination. Blood samples were collected from malaria-endemic areas, Thailand, including Mae Hong Son and Kanchanaburi provinces. *P. falciparum*-positive blood specimens were collected in an EDTA tube, and approximately 100 *μ*l of blood samples was dropped on a piece of Whatman 3M filter paper and dried (dried blood spot; DBS). Each DBS was individually stored in small plastic bags at room temperature before transporting to laboratory until the next step of the investigation.

**Figure 1:**
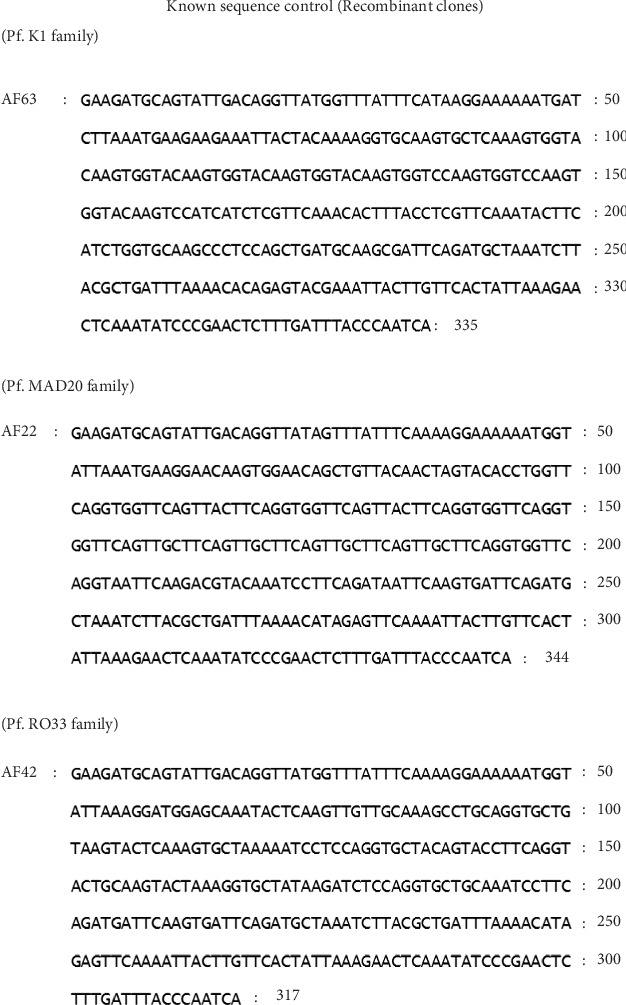
The nucleotide sequences of reference DNA (recombinant clones) of *PfMSP*1 block 2 *P. falciparum* K1, MAD20, and RO33 alleles [20].

*P. falciparum* MAD20, RO33, and K1 families reference DNA, which were recombinant plasmid, contained block 2 of *Pfmsp1* described by Ngrenngarmlert et al., [20]. The known sequence reference DNA (recombinant clones) is showed in Figure 1. The *P. falciparum* K1 culture that was kindly provided by Dr. Sumali Kamchonwongphaisan, Office of the National Science and Technology Development, Thailand, was used as monoclone *P. falciparum* control.

### 2.2. DNA Extraction

Parasite DNA was extracted from DBS using the Chelex method with some modifications [21]. Pieces of DBS were put in a microcentrifuge tube, and 300 *μ*l of sterile distilled water was added. After 10 min room temperature incubation, 300 *μ*l of supernatant was transferred to a microcentrifuge tube, and then, 1 ml of sterile distilled water was added and incubated at room temperature for 30 min. After centrifugation for 3 min at 12,000 rpm, removed the supernatant, and 6% Chelex-100 Resin (Bio-Rad Laboratories, Hercules, CA), was added and then, the tube was incubated for 30 min at 56°C and followed by incubation at 100°C for 8 min. After centrifugation for 3 min at 12,000 rpm, the supernatant of extracted DNA was kept at -20°C for long-term storage.

### 2.3. Block 2 *Pfmsp1* Gene Amplification by Nested PCR

Nested PCR amplification of *P. falciparum* DNA was carried out using the Techne TC-3000 Thermal cycler (Chemoscience, USA). The primary PCR was performed according to Snounou et al. [22]; primer pairs corresponding to blocks 1-4 regions of *Pfmsp1* gene consisted of forward primer M1-OF: 5′-CTAGAAGCTTTAGAAGATGCAGTATTG-3′ and reverse primer M1-OR: 5′-CTTAAATAGTATTCTAATTCAAGTGGATCA-3′. The PCR reaction was set up in a final volume of 20 *μ*l containing 1 *μ*l of DNA extraction, 250 nM of each primers, 10x PCR Buffer containing 1.5 mM MgCl2 (Invitrogen, USA), 125 nM of dNTPs, and 1 unit of Taq DNA Polymerase (Intron, Korea). The PCR amplification conditions were as follows: initial denaturation at 95°C for 15 min, 25 cycles of 94°C for 1 min, 50°C for 2 min, and 72°C for 2 min with the final extension at 72°C, 2 minutes. The secondary PCR was done with specific primers to the block 2 region, previously designed by Ngrenngarmlert et al., [20], and the forward and reverse primers were C1F: 5′-GAAGATGCAGTATTGACAGG-3′ and C3R: 5′-TGATTGGTTAAATCAAAGAG-3′, respectively. The conditions and PCR reaction used for the second amplification were identical to those used for the first PCR, except that amplification was conducted for 24 cycles. Then, the PCR products were determined on 1.8% agarose gel electrophoresis, visualized under a UV transilluminator after staining with ethidium bromide.

### 2.4. Development of Heteroduplex Mobility Assay (HMA) for *Pfmsp1* Allelic Diversity

#### 2.4.1. Selection of a Reference DNA to Perform Heteroduplex Mobility Assay (HMA)

In the present study, the nucleotide sequences of MSP-1 block 2 of the reference clones (K1, MAD20, and RO33) were the DNA sequences cloned in recombinant plasmids. The control DNA was amplified from *P. falciparum* K1 culture.

Three randomly selected samples (S1, S2, and S3) were used to find out a reference DNA for HMA. The nPCR products from each sample were reacted to each of the nPCR products from three references DNA (S1+MAD20, S2+MAD20, S3+MAD20, S2+RO33, S2+RO33, S2+RO33, S3+K1, S3+K1, and S3+K1).

The HMA assay consisted of 5 *μ*L of PCR amplicons containing the block 2 of *Pfmsp1* from the second reaction by nPCR, 1 *μ*l of 10x annealing buffer (1 M NaCl, 100 mM Tris-HCl, pH 7.5, 20 mM EDTA), 1 *μ*l of 6x DNA loading buffer, and 5 *μ*l of reference DNA. The HMA conditions were as follows: denature dsDNA at 98°C for 3 minutes; after denaturing, the DNA fragments were hybridized at 4°C for 15 minutes. The heteroduplex products were analyzed on 8% polyacrylamide gel electrophoresis at 10 mA for 1.30 h. Homoduplex and heteroduplex DNA bands were stained with ethidium bromide and visualized under a UV transilluminator. The selection of one reference DNA is performed by observing the most clear heteroduplex band of the reference DNA that occurred with each of the three samples.

#### 2.4.2. The Optimum Conditions to Perform Heteroduplex Mobility Assay (HMA)

The HMA conditions were tested with the reference DNA selected from the above step. The selected reference DNA and K1 DNA with proportions were 2 *μ*l/2 *μ*l and 5 *μ*l/5 *μ*l following denaturation at 98°C for 3 minutes; after denaturing, the DNA fragments were hybridized at 4°C for 15 minutes. The heteroduplex products were analyzed on a 6%, 8%, and 12% polyacrylamide gel electrophoresis at 10 mA for 1.30 h. After polyacrylamide gel electrophoresis, the heteroduplex bands were observed. The most clear of the heteroduplex band condition was selected for the next analysis.

#### 2.4.3. The Genetic Diversity of *Pfmsp1* Was Analyzed by HMA

The optimum conditions as selected by previously described studies were used to investigate for *Pfmsp*1 diversity in 8 clinical samples.

#### 2.4.4. The Interpretation of HMA

The measurement of heteroduplex mobility was presented as a mobility index (MI) as shown in the equation below. In this study, we used the *P. falciparum* K1 strain from a culture as a control to determine the heteroduplex pattern between the references DNA.

(1) $$\begin{array}{c} \text{Mobility index }\left(\text{MI}\%\right)=\left(\frac{\text{Distance from the well bottom to the midpoint of heteroduplex band}}{\text{Distance from the well bottom to the midpoint of homoduplex band}}\right)\times 100 \end{array}$$

#### 2.4.5. Validation of Established HMA

In this experiment, we used the optimum conditions of HMA for validating the genetic diversity and reliability of HMA. In addition, the samples were repeatably tested, by within run and between run for precision evaluation of the test.

1. The 10 repeatably tested Ref DNA/K1 heteroduplex by HMA was calculated for the standard deviation (SD) of MI values
2. Three repeated experiments were performed in selected 4 samples to calculate for the MI values and variants with no interpretation

### 2.5. Data Analysis

Data are displayed by MI (%) for each duplex band. The category of the band variance was set up by using the SD value of MI heteroduplex bands of reference DNA+K1 HMA for setting the interval value. The multiplicity of infection (MOI) was defined by presenting more than one band of heteroduplex or homoduplex bands. The precision of the established HMA was presented by the coefficient of variation (CV%).

## 3. Results

### 3.1. Block 2 *Pfmsp1* Gene Amplification by Nested PCR

**Figure 2:**
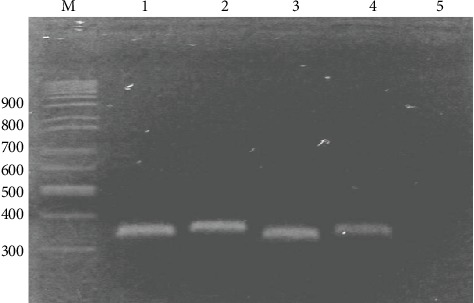
Block 2 *Pfmsp1* gene amplified by nested PCR. M: DNA marker (100 bp); lanes 1-4 were positive samples, and lane 5 was negative control.

The DNA samples subjected to *Pfmsp1* gene amplification were PCR-positive and generated around 350 bp PCR products as shown in Figure 2.

### 3.2. Optimization of HMA

**Figure 3:**
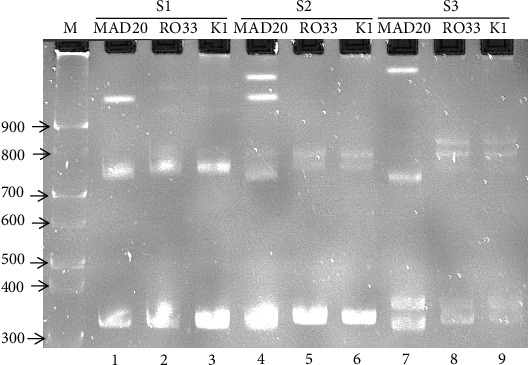
Heteroduplex Mobility Assay (HMA) between each sample and reference DNA (MAD20, RO33, K1) M: DNA marker (100 bp); lanes 1, 2, and 3: S1 sample and MAD20, R033, and K1, respectively; lanes 4, 5, and 6: S2 sample and MAD20, R033, and K1, respectively; lanes 7, 8, and 9: S3 sample and MAD20, R033, and K1, respectively.

The optimum conditions of HMA were performed by using *Pfmsp1 P. falciparum* MAD20, RO33, and K1 reference DNA annealing with random-selected three samples each (S1, S2, and S3). In Figure 3, we found that there were clear heteroduplex bands in the lane that annealing with MAD20 DNA in all samples tested. So we selected MAD20 as the reference DNA for the next step of HMA.

**Figure 4:**
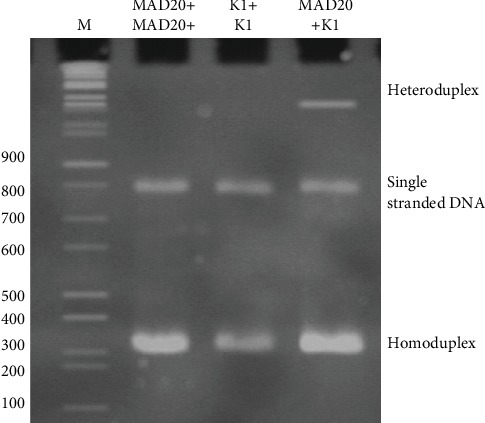
The Heteroduplex Mobility Assay (HMA), K1 (culture control)+MAD20 using 12% polyacrylamide gel with 5 *μ*l of each DNA and used electric current of 10 mA about 1 h and 30 min.

The percentages of polyacrylamide gel and DNA volume were also validated. The optimum condition of the HMA which revealed a respectable heteroduplex band was the use of 12% polyacrylamide gel with 5 *μ*l of each DNA by static electricity 10 mA for 1 h and 30 min. Then, the MAD20 reference DNA was tested with K1 culture control DNA. In Figure 4, the results showed a homoduplex 1 band in lane K1+K1 DNA and lane MAD20+MAD20 DNA with the single-stranded DNA band in each lane. Only lane MAD20+K1 DNA presented one heteroduplex band.

**Figure 5:**
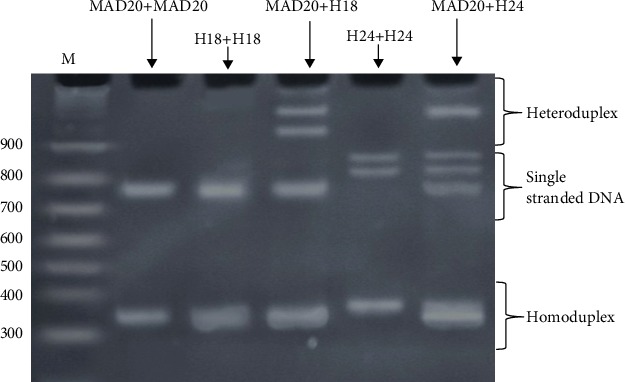
The representative gel image of the Heteroduplex Mobility Assay (HMA), MAD20+H18, and MAD20+H24 using 12% polyacrylamide gel. This figure shows homoduplex of MAD20 and each sample and heteroduplex of each sample+MAD20.

In Figure 5, the results showed the representative gel image of homoduplex and heteroduplex bands in samples H18 and H24 using MAD20 reference DNA.

### 3.3. Genetic Diversity of *Pfmsp1* Gene Block 2 by nPCR-Heteroduplex Mobility Assay (nPCR-HMA) Using MAD20 as Reference DNA

**Figure 6:**
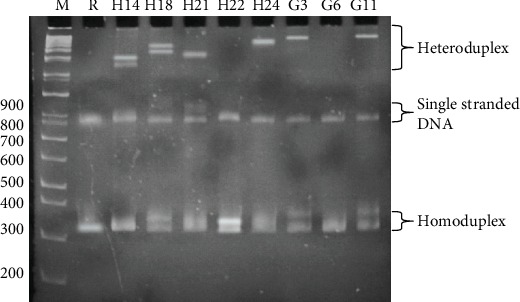
The Heteroduplex Mobility Assay (HMA) of DNA from *P. falciparum*-positive blood specimens and MAD20 reference DNA. R: MAD20 reference DNA. H14, H18, H21, H24, G3, G6, and G11 were clinical samples using MAD20 as reference DNA.

The optimum HMA conditions as mentioned above were used to validate the genetic diversity of the *Pfmsp1* block 2 gene from 8 clinical blood samples infected with *P. falciparum*. The experiment was accomplished by mixing the nPCR amplicon of *Pfmsp1* block 2 DNA from each sample with MAD20 reference DNA before denaturing and annealing. As shown in Figure 6, we found 2 heteroduplex bands that occurred in samples H14 and H18 which referred to MOI. Samples H21, H24, G3, and G11 obtained 1 heteroduplex band (single strain infection). There were two samples that did not have a heteroduplex band (H22, G6). This presented the allelic diversity of 8 allelic variants observed in different movement bands on the same PAGE. Including the 2 samples that have no heteroduplex band, we found in total 9 allele variants from 8 samples.

### 3.4. Genetic Diversity by nPCR-HMA Using MI

Next, we validated the heteroduplex band with an MI value. The movement of each band was measured to calculate MI. The results are shown in Table 1. The result revealed that the differences in MI vary from 5.32 to 17.39%.

The allelic variants were analyzed by the variation of the MI value. The result of MI values from 8 samples showed different MI accounts for 8 variants as shown in Table 1.

The 10 repeatable tested MAD20+K1 culture control heteroduplexes by HMA were calculated for the standard deviation (SD) of MI values as shown in Table 2. Then, this SD value (0.8) was used as the interval value to set up the interval scale starting from MI 5 (the round value of the lowest MI) as shown in Table 3. The results demonstrated that allelic type no.5 was the most prevalent (*n* = 5) (MI range = 8.6 − 9.4) followed by no. 1 (*n* = 3).

### 3.5. Efficiency Validation

#### 3.5.1. Reproducibility

The product of nPCR for *Pfmsp1* block2 gene of K1 and MAD20 DNA was used to validate the HMA for 10 repeats. The MI was calculated for heteroduplex DNA. The results found that MI has a variance at an acceptable coefficient of variation (CV%) that was less than 10% (CV = 5.59%) as shown in Table 4. The sampling selected 4 samples, H14, H18, G3, and G11, that were classified gene variance by triplicate MI each. The results showed the same allelic variant no. of 5/6 allelic variants (83.33%). But all samples presented the same amount of heteroduplex bands in each repeat (100%). This indicated a good reproducibility of the established nPCR-MHA as shown in Table 4.

#### 3.5.2. Accuracy Validation

This experiment was carried to validate the accuracy of HMA by using *P. falciparum* (K1) which have similarities with 77% of MAD20 DNA by nucleotide sequencing and performed the HMA between them. The results found only one heteroduplex band and one homoduplex band. The single homoduplex band found in MAD20 DNA matched to MAD20 DNA or K1 DNA match to K1 DNA. This indicates that HMA performance as theory presents the accuracy of the MHA.

## 4. Discussion

Widespread genetic diversity of *P. falciparum* in field isolates is believed to be a major complication for the development of an effective vaccine, with the increasing drug resistant and MOI detection against *P. falciparum* malaria. Therefore, a survey on genetic polymorphism of malaria parasite in clinical isolates from diverse endemic areas is required to control the disease and vaccine development. One of the most commonly used markers for genotyping of *P. falciparum* is *PfMSP-1* [20, 22, 23].

In this study, we developed nPCR-HMA using *P. falciparum* MAD20 as the reference DNA and validated it to screen *Pfmsp*1 allelic variations, since HMA has been shown to have a high efficiency for genetic variation detection as previously reported [10, 24, 25]. In addition, it is simple and more cost-efficient versus nucleotide sequencing.

The nPCR for amplifying the *Pfmsp*1 gene which has a product size of 300-400 bp succeed for all of 8 samples tested (100%). This indicates that the method has high sensitivity (100%) to amplify the *Pfmsp*1 target gene.

We have established the optimum conditions using MAD20 *P. falciparum* DNA as reference DNA and *P. falciparum* strain K1 from culture as control DNA—the use of 5 *μ*l of each nPCR product, with 98°C denature for 3 min and annealing for 15 min and then run on 12% polyacrylamide gel electrophoresis using a constant current supply of 10 mA of 1 h and 30 min. It was able to present obvious heteroduplex and homoduplex bands. This indicated that this technique can be used to separate variants of *P. falciparum* infection with convenience and rapid for a large number of samples simultaneously.

In addition, the established nPCR-HMA can discriminate the *P. falciparum* allelic variants to be 9 allelic variants from 8 clinical isolates. This clearly confirmed because each sample tested was on the same run of polyacrylamide gel electrophoresis. The results indicated that nPCR-HMA of *Pfmsp1* has good efficiency to detect the genetic diversity of *P. falciparum.*

Compared to previous researches, this study showed more rapid than the report by Kuroiwa (Kuroiwa et al., 2004), which can be excluded species of respiratory syncytial virus A for 31 alleles by using 150 V at 3 hours. In addition, Wang & Hiruki (Wang & Hiruki, 2001) differentiated the aster yellows phytoplasma group and the clover proliferation which uses voltage of 200 to 250 V, 3 to 4 hours. Nevertheless, there is a report that presented that HMA are less time-consuming than this study, such as the study of species differentiation of enteric adenoviruses by using 110 V, 75 minutes (Soares, et al., 2004). However, these depend on different DNA sizes, concentration of polyacrylamide gel, and the voltage of polyacrylamide gel electrophoresis.

This study accessible that the established nPCR-HMA found 2 MOI from 8 clinical samples (25%), which convinced the potential of the method for genotyping and showing a high MOI found in *P. falciparum* in Thailand.

The validation of the developed nPCR-HMA quality revealed a high precision, with CV (5.59%). In addition, tests also showed the same amount of band presented (100%) and also showed the same allelic no. (83.33%). The experiment of HMA between MAD20 reference DNA and K1, which is a single, strain breeding, found only 1 heteroduplex. In addition, the tests showed the test of MAD20/MAD20 or K1/K1 HMA does not have a heteroduplex band, but they presented a homoduplex band only. Our results demonstrated that a developed nPCR-HMA for the *Pfmsp1* gene has a good accuracy and precision of allelic detection.

## 5. Conclusion

We found that the established nPCR-HMA is a rapid and reproducible method that can detect heteroduplexes derived from nPCR amplicons for a *PfMSP1* block 2 target site. It can discriminate the *P. falciparum* allelic variants to be 9 allelic variants from 8 clinical isolates. This is clearly confirmed because each sample tested was on the same run of polyacrylamide gel electrophoresis. Moreover, we can classify the allelic variants by MI in a high reproducibility with a good accuracy. The results demonstrated that the established nested PCR-HMA is effective to use for screening *Pfmsp*1 allelic variations and indicated that the *P. falciparum* from clinical isolates has a high genetic diversity. These results also showed a high prevalence of the mix allelic infection.

## Figures and Tables

**Table 1:** MI values of each heteroduplex band from sample DNA and MAD20 reference DNA by HMA.

Sample no.	Numbers of heteroduplex band	MI (%)	
		Band 1	Band 2
H14	2	16.52	17.39
H18	2	8.70	10.43
H21	1	14.29	—
H22	0	—	—
H24	1	7.69	—
G3	1	5.48	—
G6	0	—	—
G11	1	5.32	—

**Table 2:** Repeatable tests of MAD20+K1culture control heteroduplex MI by HMA.

Repeatable no.	MI (%) of heteroduplex MAD20+K1
1	13.98
2	14.76
3	13.49
4	15.22
5	13.22
6	14.26
7	13.43
8	15.45
9	14.99
10	14.86
Mean	14.36
SD	0.80
CV%	5.59

**Table 3:** Allele variant categories by MI interval value.

Allele variant no.	Range of MI (%)		*N*
1	5	5.8	3
2	5.9	6.7	1
3	6.8	7.6	1
4	7.7	8.5	1
5	8.6	9.4	5
6	9.5	10.3	0
7	10.4	11.2	0
8	11.3	12.1	0
9	12.2	13	0
10	13.1	13.9	0
11	14	14.8	0
12	14.9	15.7	2
13	15.8	16.6	1
14	16.7	17.5	1
15	0		2

**Table 4:** The sampling selected 4 samples, H14, H18, G3, G11, that were classified gene variance by triplicate MI each.

Repeat no.	Sample no.	Band amount	MI (%) band 1	Allele variant no. band 1	MI (%) band 2	Allele variant no. band 2
1	H14	2	16.52	13	17.39	14
2	H14	2	15.97	13	16.96	14
3	H14	2	16.21	13	17.29	14
1	H18	2	8.70	5	10.43	7
2	H18	2	9.35	5	9.40	5
3	H18	2	8.89	5	9.05	5
1	G3	1	5.48	1	—	—
2	G3	1	5.68	1	—	—
3	G3	1	5.78	1	—	—
1	G11	1	5.32	1	—	—
2	G11	1	5.12	1	—	—
3	G11	1	5.02	1	—	—

## Data Availability

The data used to support the findings of this study are included within the article.
